# Fluid balance after cardiac arrest: Any impact on outcome? Insights from the MIMIC IV database

**DOI:** 10.1016/j.resplu.2025.101037

**Published:** 2025-07-17

**Authors:** Juliette Didier, Juliette Murris, Hélène Schopper, Emmanuelle Guérin, Nolwen Flajoliet, Marine Amiot, Bertrand Hermann, Stéphane Germain, Alain Cariou, Jean-Luc Diehl, Sandrine Katsahian, Nicolas Bréchot

**Affiliations:** a Service de Médecine Intensive-Réanimation, Hôpital Européen Georges-Pompidou, Assistance Publique-Hôpitaux de Paris (AP-HP Centre), Paris, France; b Université Paris Cité, Paris, France; c R&D, Pierre Fabre, Boulogne-Billancourt, France; d INSERM, UMR 1138, Centre de Recherche des Cordeliers, Centre Inria de Paris, Équipe HeKA, Paris, France; e Center for Interdisciplinary Research in Biology, Collège de France, CNRS-INSERM U1050, Université PSL, Paris, France; f INSERM U1266, Institute of Psychiatry and Neurosciences of Paris, Paris, France; g Service de Médecine Intensive-Réanimation, Hôpital Cochin, AP-HP Centre, Paris, France; h INSERM U1140, Innovative Therapies in Hemostasis, Paris, France; i Unité de Recherche Clinique, Hôpital Européen Georges-Pompidou, AP-HP Centre, Paris, France; j Centre d’Investigation Clinique 1418 Epidémiologie Clinique, Paris, France

**Keywords:** Cardiac arrest, Post-resuscitation, Shock, Vasoactive-inotropic score, Vascular leakage, Fluid balance

## Abstract

**Background:**

Fluid balance is closely associated with outcomes in septic shock. Post-cardiac arrest (CA) shock, which accounts for one-third of deaths after CA, shares many pathophysiological features with sepsis. However, the impact of fluid balance has not been analyzed in this setting. This study aimed to assess the impact of fluid balance on mortality during post-CA shock.

**Methods:**

This retrospective study used the Medical Information Mart for Intensive Care (MIMIC)-IV database. Fluid balance was quantified during the first 72 h. Multivariate logistic-regression analysis identified factors associated with early (by day 3) mortality.

**Results:**

Among the 1800 patients resuscitated from CA, 868 (68 %) developed post-CA shock. Of these, 801 were analyzed; 334 (42 %) died within 3 days. Early non-survivors had a higher median fluid balance (+3289 mL [25th; 75th IQR + 502; +5806]) than early survivors (+930 mL [–2677; +4353]) (*P* < 0.001). Positive fluid balance independently predicted death by day 3 (OR 2.23, 95 % CI [1.29; 3.14]; *P* = 0.005). Mortality increased significantly with increasing fluid balance, especially from 1000 mL and upwards (OR 4.11, 95 % CI [2.32; 2.47]; *P* < 0.001). Other factors independently associated with early mortality included age >66 years, maximum catecholamines dose, and peak serum lactate.

**Conclusions:**

These findings confirm that fluid balance is associated with early mortality during post-CA shock.

## Introduction

Survival post-cardiac arrest (CA), concerning 20–150 patients per 100,000 persons worldwide annually, remains <10 %.^1^, ^2^, ^3^ Among patients achieving a return of spontaneous circulation (ROSC), post-CA shock, triggered by tissue ischemia–reperfusion lesions, is responsible for one-third of secondary deaths.^4^, ^5^ Management of fluid therapy is a crucial component in treating shock, a condition characterized by increased vascular permeability. Initially described in the context of sepsis, vascular hyperpermeability arises from acute systemic inflammation following immune-cell recognition of pathogens (through pathogen-associated molecular patterns) and molecules released from damaged tissues (through damage-associated molecular patterns).^6^, ^7^ At the macrocirculation level, vascular hyperpermeability worsens circulatory failure by inducing hypovolemia, which imposes the infusion of high amounts of fluids to restore adequate hemodynamics. At the microcirculation level, fluid administration in the presence of vascular hyperpermeability results in significant capillary leakage, which is considered highly detrimental. This leakage increases interstitial pressure, worsening tissue hypoperfusion and contributing to secondary organ dysfunctions. Furthermore, administering additional fluids may raise the mean circulatory filling pressure, thereby further promoting fluid leakage from the vasculature. As a result, fluid balance (difference between fluid inputs and outputs) associates independently with mortality during sepsis.^8^, ^9^

Ischemia-reperfusion injuries to the tissues also induce severe inflammation.^10^ Thus, post-CA shock shares many pathophysiological characteristics with sepsis, e.g., comparable levels of circulating inflammatory cytokines and high amounts of fluids received by the patients.^4^, ^5^, ^11^, ^12^, ^13^, ^14^ However, the impact of fluid balance on patient outcomes has barely been analyzed to date in this particular setting. This study aimed to assess the impact of fluid balance on outcomes after CA.

## Methods

We conducted a retrospective multicenter analysis of information available in the Medical Information Mart for Intensive Care (MIMIC)-IV database. The study complied to STROBE criteria.

### Patients

The MIMIC databases are the largest open-source and free clinical databases in the critical care and emergency fields, containing a large monocenter cohort retrospectively sourced from the electronic health records of the Beth Israel Deaconess Medical Center (Boston, MA, USA). The latest version, MIMIC-IV (v2.0), contains data from 2008 to 2019.^15^ Human Ethics and Consent to Participate declarations were not applicable: The patient-information collection and research-resource creation was reviewed by the Beth Israel Deaconess Institutional Review Board that granted a waiver for informed consent and approved the data-sharing initiative. We obtained the right to use the database for this study via a dedicated form. Because data were anonymized and our study had no impact on patient management, no additional authorization was required.

The database comprises two separate modules, *hosp* and *icu*. The first concerns subsidiary data of patients recorded during their hospitalizations in other departments, while the *icu* module contains data sourced from the clinical information system (MetaVision), recorded during intensive care or emergency stays. We used the *icu* module to screen patients >18 years old successfully resuscitated from CA (with sustained ROSC). They were identified by admission annotations of “cardiac arrest” or cardiopulmonary resuscitation procedures performed in the department, excluding duplicates. Among them, we selected the patients who had developed shock within the first 24 h post-CA. Post-CA shock was defined as receiving at least one catecholamine infusion and serum lactate >2 mmol/L (>18 mg/dL) within the first 24 h after ROSC.^5^, ^16^ Patients without available fluid balances or catecholamine doses received during the first 3 days were excluded.

### Study variables and outcomes

We first assessed the impact of fluid balance on mortality during post-CA shock. The admission day, (D)0, was counted from department-admission for patients who were hospitalized right after CA resuscitation, or from the time of resuscitation for patients who had an in-intensive care unit CA, until midnight. Mortality was assessed on D3, D7 and D14, and at 1 and 3 months. Because shock-related deaths mostly occur within the first 3 days post-CA, we chose mortality by D3 as the primary endpoint for subsequent analyses.^5^, ^16^

Fluid balance was cumulated over D0–D3. Inputs were the total amount of vascular filling with crystalloids or colloids and transfusions of blood products or blood-derived products. Enteral fluids were not taken into account in our study. Given that we focused on the very early phases after cardiac arrest, their contribution to the fluid balance was considered marginal and subject to significant discrepancies between prescribed and actually received amounts.^17^ Outputs were quantified as diuresis, ultrafiltration and digestive losses collected by gastric tubes or proximal ileostomies. To characterize extents of post-CA shock and hemodynamic failure, the type and dose of each catecholamine received during the first 72 h was recorded. That information enabled calculation of the “Vasoactive-Inotropic Score” (VIS), as follow: VIS = 10,000 × vasopressin dose (U/kg/min) + 100 × epinephrine dose (μg/kg/min) + 100 × norepinephrine dose (μg/kg/min) + 50 × levosimendan dose (μg/kg/min) + 25 × olprinone dose (μg/kg/min) + 20 × methylene blue dose (mg/kg/min) + 10 × milrinone dose (μg/kg/min) + 10 × phenylephrine dose (μg/kg/min) + 10 × terlipressin dose (μg/min) + 0.25 × angiotensin-II dose (ng/kg/min) + dobutamine dose (μg/kg/min) + dopamine dose (μg/kg/min) + enoximone dose (μg/kg/min).^18^, ^19^ The mean daily VIS for each patient (D0, D1, D2 and D3) was calculated, and then the highest value was called the “maximum mean VIS”, with each day being divided into calendar days from midnight to midnight.

Finally, all available demographic data, hemodynamic monitoring, variables reflecting the severity of organ failure and treatments received were also collected. Unfortunately, intra-arrest informations (initial recorded rhythm, duration of no- and low-flow…) are not available in this database. Also, neurological outcomes could not be assessed.

### Statistical analyses

Quantitative variables are expressed as means or medians [25th; 75th interquartile ranges] and compared with Student’s *t*-test. Qualitative variables are reported as numbers (percentages) and compared with analysis of variance (ANOVA) or Fisher’s exact test. Risk factors for death post-CA shock were identified using univariate and multivariate models. Because fluid balance was identified as a non-linear factor in the analysis of associated covariates of vital status on D3, a thorough analysis was conducted to identify thresholds to categorize it. The selected approach minimized the Akaike Information Criterion and was based on tuned decision trees.^20^ Sensitivity analyses applied a categorization based on clinical expertise (positive and negative values). Categorization of other variables relied on their medians and quartiles. All those variables were subjected to pairwise Pearson correlation tests that selected only one among strongly correlated variables, to prevent model instability due to autocorrelation. In addition, variables with missing values exceeding 10 % were removed from the multivariate models.

For each outcome (death by D3, D7 and D14, and 1 and 3 months), multivariate logistic-regression models including all the variables were computed. These models were then compared to regressions including only variables that had achieved *P* < 0.2 in univariate logistic-regression analyses, with no additional variable selection thereafter. The final models were chosen based on the Akaike Information Criterion, taking the lowest value among models computed for the entire study population. Associations between various variables and death at different times are reported as odds ratios (OR) [95 % confidence intervals (CI)].

Overall survival was estimated with the Kaplan–Meier method and compared with log-rank tests between patients with positive or negative fluid balances over D0–D3, and each VIS quartile. The significance threshold was set at 0.05, and all tests were two-tailed.

Statistical analyses were computed with R software (version 4.2.0, R Core Team 2022, R Foundation for Statistical Computing, Vienna, Austria).

## Results

### Study population

**Fig. 1:**
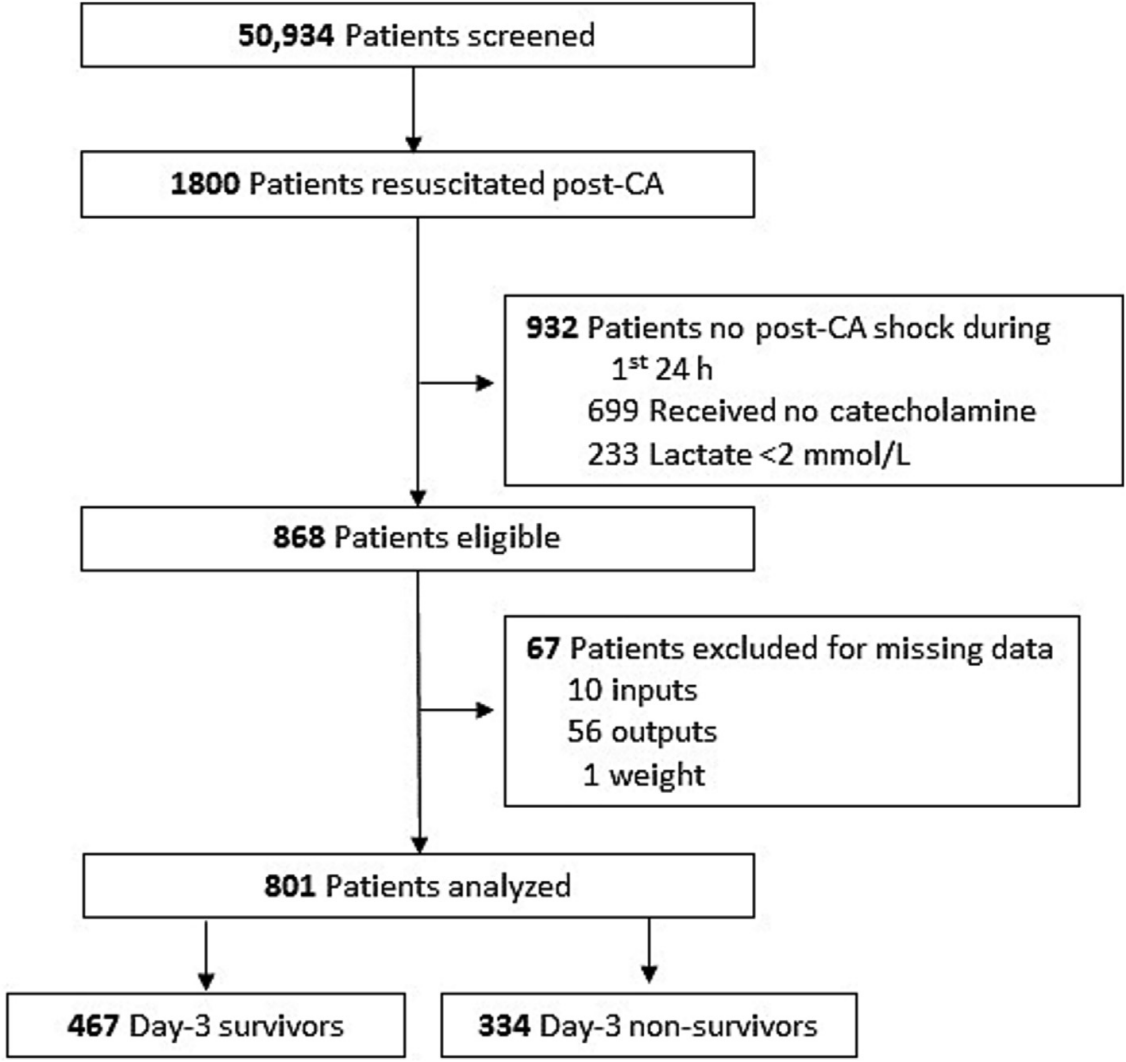
Flow chart of the study.

Among the 50,934 patients screened between 2008 and 2019, 1800 achieved sustained ROSC post-CA. Among them, 868 (48 %) developed post-CA shock and 801 were retained for the analyses (Fig. 1). Post-CA shock severity was confirmed by 41.7 % mortality by D3.

Patients’ detailed characteristics are reported in Table 1. Most patients were men (60.8 %) and median age was 66 years. Post-CA shock was severe for most patients. The median maximum VIS was 25.6 µg/kg/min, with 202 patients receiving very high catecholamine doses, with maximum VIS > 55 µg/kg/min. The median D0–D3 lactatemia peak was 5.8 mmol/L. Patients received high amounts of fluids, which translated into a median D0–D3 fluid balance of +1847 [–1459; +5062] mL.

**Table 1:** Characteristics of day-3 survivors and non-survivors of post-CA shock.

Characteristic	Total population *N* = 801	D3 survivors *N* = 467	D3 non-survivors *N* = 334	*P* value
Demographics				
Age	66 [54; 77]	66 [56; 77]	66 [52; 77]	0.329
Male sex	487 (60.8)	285 (61.0)	202 (60.5)	0.875
BMI (*n* = 549/375 alive/174 dead)	27 [24; 32]	28 [24; 32]	27 [23; 32]	0.201
Chronic Liver failure	3 (0.4)	1 (0.2)	2 (0.6)	0.574
Chronic kidney failure	116 (14.5)	80 (17.1)	36 (10.8)	**0.012**
History of cancer	72 (9)	33 (7.1)	39 (11.7)	**0.024**
Chronic respiratory disease	45 (5.6)	26 (5.6)	19 (5.7)	0.941
In-ICU				
At admission				
Serum pH (*n* = 669)	7.37 [7.29; 7.40]	7.37 [7.33; 7.41]	7.29 [7.19; 7.35]	**<0.001**
SAPS-II (*n* = 379)	72 [69; 75]	71 [69; 75]	72 [69; 75]	**0.005**
GCS (*n* = 732)	3 [3;11]	4 [3; 12]	3 [3;9]	**0.012**
Peak lactatemia D0-D3 (mmol/L)	5.8 [3.6; 9.5]	4.7 [3.1; 7.5]	8.2 [5.3; 12.3]	**<0.001**
Maximum mean VIS* D0–D3 (µg/kg/min)	26 [11; 55]	17 [8; 30]	51 [25; 91]	**<0.001**
<12	213 (26.6)	174 (37.3)	39 (11.7)	**<0.001**
[12–26]	194 (24.2)	145 (31.0)	49 (14.7)	
[26–55]	192 (24)	103 (22.1)	89 (26.6)	
>55	202 (25.2)	45 (9.6)	157 (47.0)	
Fluid balance D0–D3 (mL)	1,847 [–1,459; 5,062]	930 [–2,677; 4,353]	3,289 [502; 5,806]	**<0.001**
Negative	266 (33.2)	214 (45.8)	52 (15.6)	
Low [0–1000]	100 (12.5)	41 (8.8)	59 (17.7)	
Medium [1000–7000]	298 (37.2)	135 (28.9)	163 (48.8)	
High ≥ 7000	137 (17.1)	77 (16.5)	60 (18.0)	
Targeted temperature management	285 (35.6)	149 (31.9)	136 (40.7)	**0.011**
Dialysis	122 (15.2)	83 (17.8)	39 (11.7)	**0.018**
PaO_2_/FiO_2_ (*n* = 740)	160 [93; 270]	184 [115; 285]	131 [80; 252]	**<0.001**
VA-ECMO	11 (1.4)	8 (1.7)	3 (0.9)	0.376
Days in ICU	6.0 [1.3; 7.5]	9.3 [4.1; 11.1]	1.4 [0.6; 2.1]	**<0.001**

Values are expressed as median [25th; 75th IQR] or *n* (%).

BMI, body mass index. D, day; GCS, Glasgow coma scale; ICU, intensive care unit; SAPS, simplified acute physiology score; VA-ECMO, venoarterial extracorporeal membrane oxygenation; VIS, vasoactive-inotropic score.

* Calculated as in 19.

### Factors associated with early (by D3) mortality

**Fig. 2:**
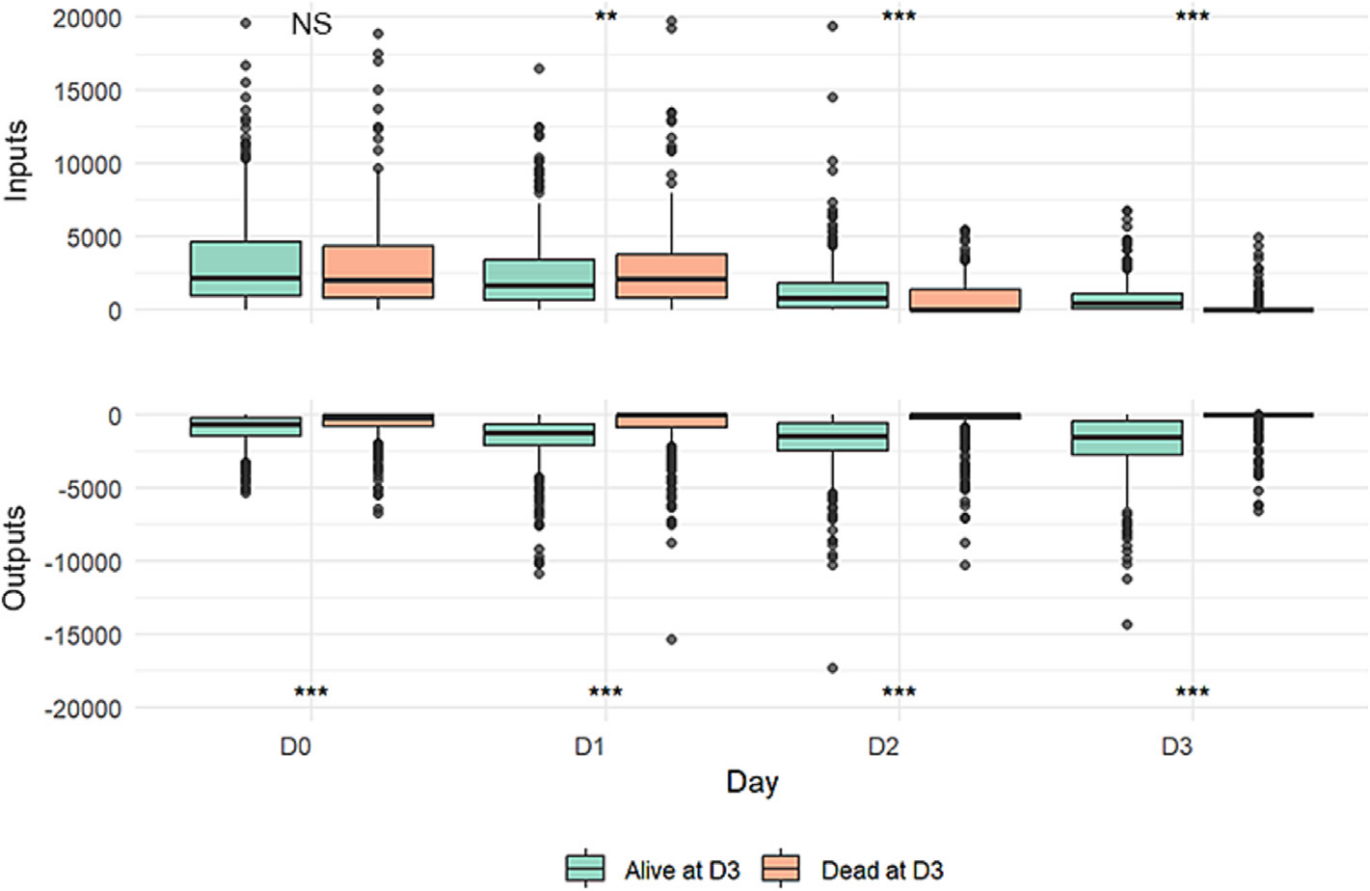
Tukey’s box plots of detailed fluid inputs and outputs in mL over the first 3 days post-cardiac arrest, of day-3 survivors and non-survivors. NS, non-significant. **P < 0.01, ***P < 0.001.

D3 non-survivors, compared to survivors beyond D3, had more severe post-CA shock (Table 1). They had more severe hyperlactatemia, and received higher catecholamine doses. Despite higher amounts of fluid infused the first 2 days, their urine outputs were smaller, leading to a fluid balance of + 3289 [+502; +5806] mL vs. +930 [–2677; +4353] mL respectively, *P* < 0.001 (Fig. 2). The mean fluid balance over 3 days (accounting for early death or ICU discharge) confirmed a higher fluid balance in non-survivors (4307 ± 6949  ml vs. 123 ± 1454  ml in survivors, *p* < 0.01 between groups). Fluids mostly consisted of normal saline (71 % of fluids received), followed by balanced cristalloids (21 %), colloids (3 %) and blood products (4 %). None of these proportions differed significantly between survivors and non-survivors at day 3. Interestingly, a much higher proportion of survivors received diuretics during this period (62.5 % vs. 19.7 %, p < 0.001). Although this result may largely reflect their better hemodynamic status, it may also have contributed to a lower fluid balance in survivors.

Univariate analysis of predictors of death by day 3 identified age <66 years old and chronic kidney failure as protective factors, while post-CA shock severity, reflected by the peak lactatemia level, maximum VIS and fluid-balance level, associated with early mortality (Table 2).

**Table 2:** Parameters associated with day-3 mortality after post-CA shock.

Parameter	Univariate analysis		Multivariate analysis	
	OR [95 % CI]	*P* value	OR [95 % CI]	*P* value
Age (years)				
<54	Reference	–	–	
[54–66]	0.57 [0.38; 0.86]	**0.007**	0.51 [0.26; 0.97]	**0.041**
[66–77]	0.72 [0.48; 1.07]	0.101	0.43 [0.22; 0.83]	**0.014**
>77	0.78 [0.52; 1.15]	0.208	0.68 [0.35; 1.33]	0.260
BMI				
[18–25]	Reference	–	–	
[25–30]	0.80 [0.51; 1.25]	0.322	0.87 [0.48; 1.55]	0.633
[30–35]	0.74 [0.44; 1.23]	0.250	1.21 [0.62; 2.36]	0.578
<18	0.60 [0.13; 2.10]	0.455	0.33 [0.05; 1.63]	0.198
≥35	0.77 [0.43; 1.36]	0.377	0.93 [0.44; 1.93]	0.838
History of cancer	1.74 [1.07; 2.84]	**0.026**	0.98 [0.43; 2.17]	0.961
Chronic Kidney failure	0.58 [0.38; 0.88]	**0.012**	0.48 [0.21 1.08]	0.083
Peak lactatemia D0–D3 (mmol/L)	1.20 [1.16; 1.25]	**<0.001**	1.13 [1.07; 1.21]	**<0.001**
Maximum mean VIS* D0–D3 (µg/kg/min)				
<12	Reference	–	–	
[12–26]	1.51 [0.94; 2.43]	0.090	1.50 [0.74; 3.11]	0.267
[26–55]	3.86 [2.48; 6.09]	**<0.001**	3.53 [1.76; 7.36]	**0.001**
>55	15.57 [9.74; 25.46]	**<0.001**	7.94 [3.68; 17.88]	**<0.001**
Fluid balance D0–D3 (mL)				
≤0	Reference	–	–	
[0–1000]	5.92 [3.61; 9.84]	**<0.001**	4.09 [1.87; 9.04]	**<0.001**
[1000–7000]	4.97 [3.42; 7.31]	**<0.001**	4.11 [2.32; 7.47]	**<0.001**
≥7000	3.21 [2.04; 5.07]	**<0.001**	1.15 [0.55; 2.39]	0.707
Targeted temperature management	1.47 [1.09; 1.96]	**0.010**	1.34 [0.83; 2.15]	0.231
Dialysis	0.61 [0.40; 0.92]	**0.019**	0.57 [0.28; 1.14]	0.116
PaO_2_/FiO_2_				
≥300	Reference	–	–	
[150–300]	0.78 [0.51; 1.21]	0.270	0.72 [0.36; 1.43]	0.345
<150	1.74 [1.18; 2.61]	**0.006**	1.25 [0.65; 2.42]	0.507

Values are expressed as median [25th–75th IQR] or *n* (%).

BMI, body mass index; D, day; VIS, vasoactive-inotropic score.^19^

* Calculated as in 19.

**Fig. 3:**
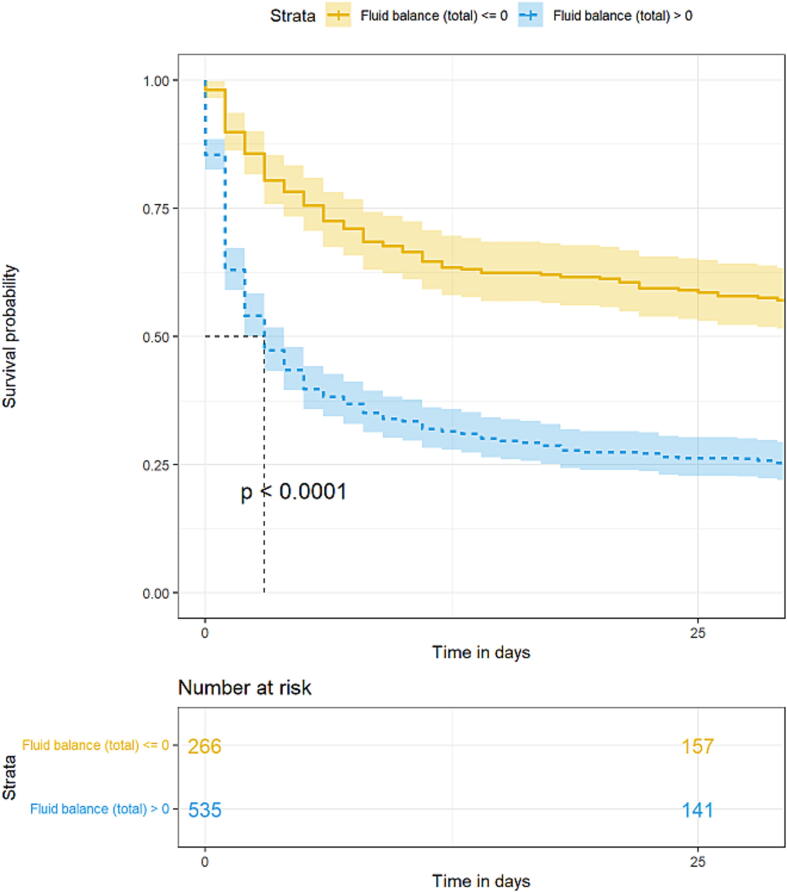
Kaplan–Meier estimated survival probability according to cumulative fluid balance over days 0–3 of post-cardiac arrest shock, with shaded 95 % confidence interval.

After adjusting for shock severity (maximum VIS, lactatemia) and other factors associated with early death according to logistic-regression analysis, a positive total fluid balance remained an independent predictor of death by D3 (Table 2 and Fig. 3). Among patients with a positive fluid balance, mortality rose significantly with increasing balance, especially as of +1000 mL and higher (OR 4.11 [95 CI 2.32; 2.47]; *P* < 0.001). The D3-mortality rate was 16 % for patients with a negative fluid balance, compared to 46 % for those with a positive balance (OR 2.23 [95 % CI 1.29; 3.14]; *P* = 0.005). Importantly, the association between fluid balance over D0–D3 and mortality persisted at later times (Fig. 3). Analyzing more precisely outputs vs inputs in the cohort, we identified a subgroup of patients with a significant profile of low outputs despite high inputs (Supplementary Appendix 1). This subgroup comprised patients exhibiting much greater hemodynamic compromise compared to the rest of the cohort, with higher doses of catecholamines administered, higher peak lactate levels, and more severe acidosis (Supplementary Appendix 2). Interestingly, almost all patients in this group died (91 %). Accordingly, this group was significantly overrepresented among non-survivors compared to survivors (14 % vs. 1 %, p < 0.0001, Fisher’s exact test). Other ‘profiles’ (low outputs & low inputs, high outputs & low inputs, and high outputs & high inputs) were equally distributed between survivors and non-survivors. Intriguingly, very high fluid-balance levels, exceeding +7000 mL, seen for 137 patients, did not significantly impact early mortality according to our multivariate analyses (Table 2). These patients were younger than the entire cohort, with more pronounced hemodynamic impairment and multiorgan failure at admission (Supplementary Appendix 3). Although their D3 mortality was comparable to that of the entire cohort (44 % vs. 41 %; *P* = 0.63), by D30 it was higher (74 % vs. 62 %, respectively; *P* = 0.006).

**Fig. 4:**
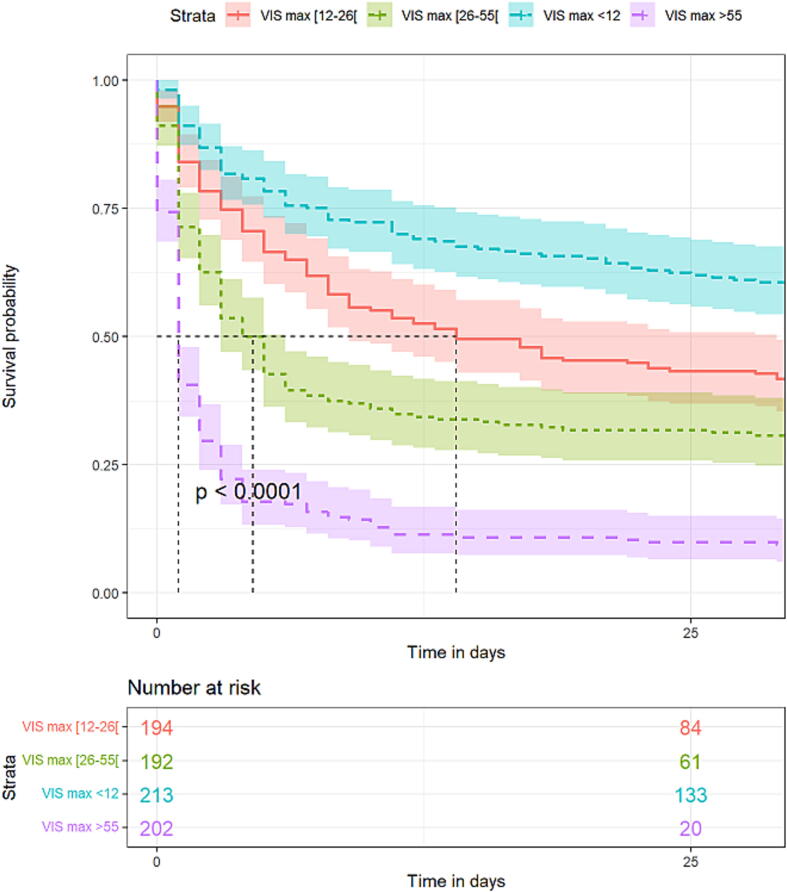
Kaplan–Meier estimated survival probability according to maximal vasoactive-inotropic score (VIS) over days 0–3 of post-cardiac arrest shock, with shaded 95 % confidence interval.

Other factors independently associated with D3 mortality were age >66 years and post-CA shock severity. Indeed, the maximum catecholamine dose received by the patients was associated with early death, with a maximum VIS > 55 µg/kg/min multiplying the risk of dying by almost × 8 (Table 2 and Fig. 4). Peak lactatemia was also an independent predictor of D3 mortality. Each additional lactatemia (mmol/L) point translated into a 1.13× increased risk of mortality by D3 (*P* < 0.001).

## Discussion

This novel study enabled us to identify an independent association between D0–D3 fluid balance and early mortality of patients with post-CA shock. A fluid balance exceeding +1000 mL over that period was associated with a risk of dying multiplied by 4. Importantly, that relationship was independent of the catecholamine dose received. That finding is in accordance with observations made during septic shock, and with recent data from another group.^8^, ^21^ They are further supported by the pathophysiology of post-CA shock.^9^ Indeed, post-CA shock is a very complex hemodynamic situation, combining features of cardiogenic shock and those of distributive shock, e.g., vasoplegia and hypovolemia.^5^, ^11^ Ischemia-reperfusion tissue injuries, through the release of necrotic cell fragments (membrane, DNA, histones, …), recognized as damage-associated molecular patterns by inflammatory cells, initiate an immune process sharing many similarities with sepsis.^22^ For example, post-CA patients have high levels of circulating cytokines, comparable to those observed during sepsis, which are associated with post-CA mortality.^23^, ^12^, ^13^, ^14^ However, to date, little attention has been paid to the specific impact of fluid balance after CA.

Post-CA shock accounts for approximately one-third of post-CA deaths.^5^, ^16^ Our finding of a significant association between fluid balance and D3 mortality may suggest an important role for vascular leakage, among other factors, in this condition. Although D3 non-survivors received higher amounts of fluids during the first 2 days, that enhanced volume did not translate into comparable urine outputs compared to survivors. This is further supported by our identification of a group of patients in this cohort with low fluid outputs despite high inputs, who exhibited more severe hemodynamic compromise and extremely high mortality by day 3 (91 %). Ischemia-reperfusion, through the inflammation it induces and reactive oxygen species it releases, greatly increases vascular permeability within minutes, which might be highly detrimental.^10^ Indeed, it might further aggravate circulatory failure, via the hypovolemia it causes. Fluids needed to restore hemodynamics, administered in patients with vascular hyperpermeability, further worsen vascular leakage. Extravasated fluids increase interstitial hydrostatic pressure, and might therefore contribute to secondary organ dysfunctions, particularly transient myocardial dysfunction typical of post-CA patients.^24^ Indeed, experimental study results showed that increased water content in the heart, even as small as 4 %, dramatically impacted cardiac function.^25^ Likewise, post-CA cerebral edema, described in patients and animals, may markedly affect patients’ neurological outcomes.^26^, ^27^ This critical role of vascular leakage post-CA is also supported by animal studies, in which specifically targeting vascular hyperpermeability was highly beneficial.^28^, ^29^, ^30^ Notably, infusion of the fibrin-derived β_15–42_ fragment, which stabilizes interendothelial cell junctions after ischemia–reperfusion, strongly lowered the fluid-loading requirement in a pig model of CA, and significantly improved the animals’ neurological outcomes.^30^ Other factors impacting the fluid balance other than vascular hyperpermeability might have accounted in the association described here. Particularly, because fluid management was not standardized, some heterogeneous fluid-therapy application, leading to fluid overload, might have affected the fluid-balance association with patients’outcomes.

Our findings thus highlight the impact that fluid balance may have after CA, which could have important repercussions in clinical practice. First, it may highlight the importance of restricting fluid therapies post-CA. Although fluid expansion is necessary to counteract hypovolemia and to maintain adequate organ perfusion, fluid overload may be deleterious.^31^, ^32^ Monitoring of patient’s hemodynamic status, especially guiding fluid expansion based on the patient’s pre-load dependency, may play a particular role in this setting.^33^ A restrictive fluid strategy may explain the relative protective effect of chronic kidney disease that we observed herein, as clinicians are usually reluctant to infuse high fluid volumes into patients with advanced renal insufficiency. Further studies will be needed to better define how to optimally manage fluids in the specific setting of post-CA.

Second, given the high amounts of fluids administered to patients, our results highlight the impact that the type of fluid may have. Balanced solutions were compared to physiological saline for critically ill patients in several randomized trials. Although none of them demonstrated a clear advantage of balanced solutions, a meta-analysis of their individual data found a posterior probability of 89.5 % that balanced solutions lowered mortality in those trials.^34^ Moreover, a post-hoc analysis of the BASICS trial data revealed a protective effect of balanced solutions against mortality of septic shock patients receiving high amounts of fluids.^35^ To our knowledge, apart from a small randomized trial suggesting less pronounced acidosis in post-CA patients receiving balanced solutions compared to physiological saline, no study has addressed this important question in the specific setting of CA.^36^ In our study, patients mostly received normal saline. Whether better outcomes could be achieved using other types of fluid therapy, particularly balanced solutions, should be addressed in future studies.

Third, beyond the way to attenuate its consequences, we think our results may indicate that therapies dedicated to preventing post-CA vascular hyperpermeability are urgently needed, as it is recognized for sepsis.^37^

Apart from the fluid-balance results, the other striking observation derived from our analysis is the strong association found between the catecholamine-dose received, reflected by the VIS, and early mortality. Indeed, VIS was independently associated with D0–D3 mortality in this database population, with a clear dose–response relationship. Patients receiving catecholamines with a VIS > 26 µg/kg/min had higher risk of mortality, reaching almost 8-fold for patients with VIS > 55 µg/kg/min. Although the results of several studies highlighted the VIS impact during septic and cardiogenic shock, that association has not been previously described for post-CA patients.^19^ That finding strongly reinforces the impact of post-CA shock and its severity on overall mortality post-CA.^16^ Beyond this association, it raises questions about the optimal catecholamine-use strategy in this specific post-CA setting. Indeed, the results of a recent retrospective study suggested an increase of mortality with epinephrine versus norepinephrine use post-CA.^38^ Whether vasopressor alternatives to catecholamines, like angiotensin II, could have beneficial effects, while reducing catecholamine doses, may also merit investigation.^39^ Independence between the fluid-balance level and VIS herein reinforced the fact that, although participating in the same global process, they might be due to distinct harmful mechanisms. Overall, our results highlight the need for further studies to help guide treatment of post-CA distributive shock.

### Limitations

The major strength of our analysis resides in its large sample size, with more than 800 patients analyzed. However, it suffers also from important limitations. Because of the MIMIC-IV database constitution, all Utstein criteria were not available.^40^ Although their absence does not change the fluid balance–early mortality association identified herein, it precludes any further examination of risk factors predicting a positive fluid balance. Likewise, precise and reliable myocardial function evaluation was not available in the database. However, given the particular hemodynamic profile of cardiogenic shock patients (low cardiac output despite high intravascular volume and very high mortality), this limitation mainly leads to a risk of underestimating the association between fluid balance and mortality in our study (i.e., patients dying despite receiving relatively low fluid loading due to high intravascular volume). The rate withdrawal of active therapeutics could also not be recovered from the charts. Although this is quite uncommon at early phases of post-CA shock,^16^ it may have also contributed to an underestimation of the association described (i.e., patients not receiving the quantity fluids their hemodynamic condition would have indicated). Accordingly, the mean fluid balance over the period (accounting for early death) remained significantly higher in non-survivors than in survivors in our study. The level of therapeutic engagement may have also contributed to the failure to find a clear association between fluid balance and mortality for patients with the highest fluid balance levels (≥7000 mL) over D0–D3 in multivariate analyses, albeit selected in univariate analyses. Those patients, significantly younger and more severely ill than the rest of the population, may indeed have benefited from more intensive therapeutic intervention. That hypothesis is supported by their higher D30 mortality. Lastly, we could not extract from the MIMIC-IV database any information about patients’ long-term neurological status, which markedly affects their long-term quality of life.^41^ Given the potential role of cerebral edema in post-CA damage, evaluating the impact of fluid balance on the long-term neurological outcome of the patients will be critically important.

## Conclusions

In this observational study of 801 patients with post-CA shock, fluid balance was closely associated with day 0–3 mortality. In particular, patients exhibiting low outputs despite high inputs experienced significantly poorer survival.

## Availability of data and material

MIMIC-IV is a publicly available database sourced from the electronic health record of the Beth Israel Deaconess Medical Center. The additional datasets used and/or analyzed during the current study are available from the corresponding author on reasonable request.

## Funding sources

This research did not receive any specific grant from funding agencies in the public, commercial, or not-for-profit sectors.

## CRediT authorship contribution statement

**Juliette Didier:** Writing – review & editing, Writing – original draft, Validation, Investigation, Formal analysis, Data curation, Conceptualization. **Juliette Murris:** Writing – review & editing, Writing – original draft, Validation, Software, Resources, Methodology, Investigation, Formal analysis, Data curation, Conceptualization. **Hélène Schopper:** Software, Resources, Formal analysis, Data curation. **Emmanuelle Guérin:** Writing – review & editing, Validation, Formal analysis. **Nolwen Flajoliet:** Writing – review & editing, Validation, Formal analysis. **Marine Amiot:** Writing – review & editing, Validation, Formal analysis. **Bertrand Hermann:** Writing – review & editing, Visualization, Validation, Formal analysis. **Stéphane Germain:** Writing – review & editing, Validation, Formal analysis. **Alain Cariou:** Writing – review & editing, Validation, Formal analysis. **Jean-Luc Diehl:** Writing – review & editing, Validation, Formal analysis, Conceptualization. **Sandrine Katsahian:** Writing – review & editing, Visualization, Validation, Supervision, Software, Resources, Project administration, Methodology, Formal analysis, Data curation, Conceptualization. **Nicolas Bréchot:** Writing – review & editing, Writing – original draft, Validation, Project administration, Methodology, Investigation, Formal analysis, Conceptualization.

## Ethics approval and consent to participate

Human Ethics and Consent to Participate declarations were not applicable: the Beth Israel Deaconess Institutional Review Board that granted a waiver for informed consent and approved the data-sharing initiative reviewed the patient-information collection and research-resource creation. We obtained the right to use the database for this study via a dedicated form. Because data were anonymized and our study had no impact on patient management, no additional authorization was required.

## Declaration of competing interest

The authors declare the following financial interests/personal relationships which may be considered as potential competing interests: ‘Dr Bréchot participates in an F4-Pharma advisory board, without any financial competing interest. He received a grant from the French Ministry of Health for a study evaluating FX06, a drug under development aiming at controlling vascular leakage. He receives fees from Findimmune and Getinge, outside the scope of this study.’.
