# Supplementary figures and images for: Fluid balance after cardiac arrest: Any impact on outcome? Insights from the MIMIC IV database
Source: Resusc Plus. 2025 Jul 17;25:101037. doi: 10.1016/j.resplu.2025.101037 (PMC12329095; doi:10.1016/j.resplu.2025.101037)

**Supplementary Figure 1.** Mean outputs versus inputs over a three-day period.

**
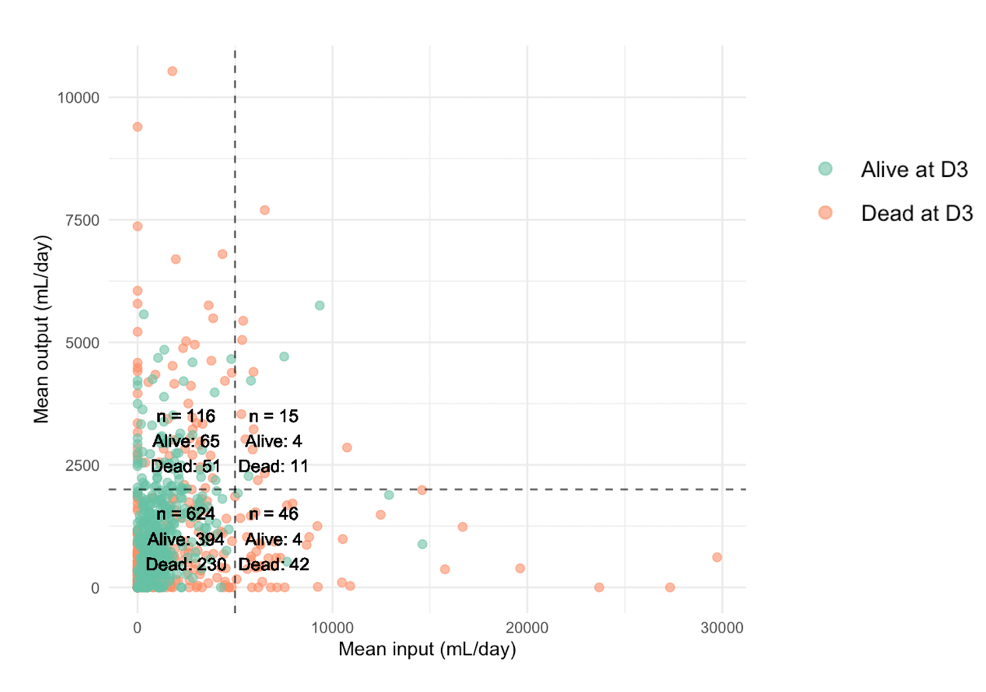
**

Supplement: Supplementary Appendix 1 [file mmc1.docx]
