# Supplementary material for: Fluid balance after cardiac arrest: Any impact on outcome? Insights from the MIMIC IV database
Source: Resusc Plus. 2025 Jul 17;25:101037. doi: 10.1016/j.resplu.2025.101037 (PMC12329095; doi:10.1016/j.resplu.2025.101037)
Supplement: Supplementary Appendix 2 [file mmc2.docx]

**Supplementary Table 1.** Characteristics of the subgroup “Low Output / High Input” (Lo/Hi)

| **Characteristic** | **Total population**  **N=801** | **Subgroup Lo/Hi**  **N=46** | **Others**  **N=755** | ***P* value** |
| --- | --- | --- | --- | --- |
| Demographics |  |  |  |  |
| Age | 66 [54; 77] | 74 [54; 80] | 66 [54; 76] | 0.13 |
| Male sex | 487 (60.8) | 26 (56.5) | 461 (61.1) | 0.54 |
| BMI (n=549; 17/532) | 27 [24; 32] | 28 [25; 30] | 27 [24; 32] | 0.67 |
| Chronic Liver failure | 3 (0.4) | 3 (0.4) | 0 (0.0) | 1.00 |
| Chronic kidney failure | 116 (14.5) | 42 (91.3) | 643 (85.2) | 0.39 |
| History of cancer | 72 (9) | 7 (15.2) | 65 (8.6) | 0.18 |
| Chronic respiratory disease | 45 (5.6) | 1 (2.2) | 44 (5.8) | 0.51 |
| In-ICU |  |  |  |  |
| At admission |  |  |  |  |
| Serum pH (n=669) | 7.37 [7.29; 7.40] | 7.21 [7.15; 7.31] | 7.36 [7.30; 7.41] | **<0.01** |
| SAPS-II (n=379) | 72 [69; 75] | 72 [69; 75] | 72 [69; 75] | 0.48 |
| GCS (n=732) | 3 [3;11] | 6 [3; 14] | 3 [3;11] | **<0.01** |
| Peak lactatemia D0-D3 (mmol/L) | 5.8 [3.6; 9.5] | 9.2 [6.2; 13.0] | 5.6 [3.5; 9.3] | **<0.01** |
| Maximum mean VIS* D0–D3 (µg/kg/min) | 26 [11; 55] | 67 [44; 94] | 23 [11; 53] | **<0.01** |
| <12 | 213 (26.6) | 2 (4.4) | 211 (28.0) | **<0.01** |
| [12–26[ | 194 (24.2) | 3 (6.5) | 191 (25.3) |  |
| [26-55[ | 192 (24) | 15 (32.6) | 177 (23.4) |  |
| >55 | 202 (25.2) | 26 (56.5) | 176 (23.4) |  |
| Fluid balance D0–D3 (mL) | 1,847 [–1,459; 5,062] | 7,854 [6,227; 11,190] | 1,123 [–1,484; 4,281] | **<0.01** |
| Negative | 266 (33.2) | 0 (0.0) | 266 (35.2) | **<0.01** |
| Low [0–1000] | 100 (12.5) | 0 (0.0) | 100 (13.3) |  |
| Medium [1000–7000[ | 298 (37.2) | 15 (32.6) | 283 (37.5) |  |
| High ≥7000 | 137 (17.1) | 31 (67.4) | 106 (14.0) |  |
| Targeted temperature management | 285 (35.6) | 11 (23.9) | 274 (23.9) | 0.11 |
| Dialysis | 122 (15.2) | 83 (17.8) | 116 (15.4) | 0.83 |
| PaO_2_/FiO_2_ (n=740) | 160 [93; 270] | 101 [70; 156] | 166 [96; 273] | **<0.01** |
| VA-ECMO | 11 (1.4) | 1 (2.2) | 10 (1.3) | 0.48 |
| Days in ICU | 6.0 [1.3; 7.5] | 1.3 [0.9; 1.6] | 6.2 [1.4; 7.9] | **<0.01** |
| 3-day mortality | 334 (41.6) | 42 (91.3) | 292 (38.6) | **<0.01** |

Values are expressed as median [25th; 75th IQR] or n (%). *Calculated as in [18]. BMI, body mass index. D, day; GCS, Glasgow coma scale; ICU, intensive care unit; SAPS, simplified acute physiology score; VA-ECMO, venoarterial extracorporeal membrane oxygenation; VIS, vasoactive-inotropic score.
