# Supplementary material for: Fluid balance after cardiac arrest: Any impact on outcome? Insights from the MIMIC IV database
Source: Resusc Plus. 2025 Jul 17;25:101037. doi: 10.1016/j.resplu.2025.101037 (PMC12329095; doi:10.1016/j.resplu.2025.101037)
Supplement: Supplementary Appendix 3 [file mmc3.docx]

**Supplementary Table 2.** Comparison of patient’s characteristics according to D0–D3 fluid balance

|  |  |  |  |  |
| --- | --- | --- | --- | --- |
| **Characteristic** | **Fluid balance** | | ***P* value** |  |
|  | **<7000 mL (n=664)** | **≥7000 mL (n=137)** |  |  |
| Demographics |  |  |  |  |
| Age | 66 [55; 77] | 62 [49; 76] | **0.032** |  |
| Male sex | 399 (60) | 88 (64) | 0.39 |  |
| BMI (n=549) |  |  | 0.53 |  |
| <18 | 12 (2.6) | 0 |  |  |
| [25–30[ | 149 (33) | 30 (32) |  |  |
| [30–35[ | 93 (20) | 17 (18) |  |  |
| [18–25[ | 136 (30) | 29 (31) |  |  |
| >35 | 66 (14) | 17 (18) |  |  |
| Chronic liver failure | 3 (1) | 0 | 1 |  |
| Chronic kidney failure | 98 (15) | 18 (13) | 0.69 |  |
| History of cancer | 58 (9) | 14 (10) | 0.62 |  |
| Chronic respiratory disease | 39 (6) | 6 (4) | 0.68 |  |
| In-ICU criteria |  |  |  |  |
| At admission |  |  |  |  |
| Serum pH (n=669) | 7.36 [7.30; 7.41] | 7.31 [7.24; 7.37] | **<0.001** |  |
| SAPS-II (n=379) | 71 [69; 75] | 72 [70; 75] | **0.008** |  |
| GCS (n=732) | 3 [3; 11] | 5 [3; 12] | 0.056 |  |
| Peak lactatemia D0–D3 (mmol/L) | 5.4 [3.4; 8.8] | 8.5 [5.3; 12.0] | **<0.001** |  |
| Maximum mean VIS* D0–D3 (µg/kg/min) |  |  | **<0.001** |  |
| <12 | 198 (30) | 15 (11) |  |  |
| [12–26[ | 169 (25) | 25 (18) |  |  |
| [26–55[ | 148 (22) | 44 (32) |  |  |
| >55 | 149 (22) | 53 (39) |  |  |
| Targeted temperature management | 250 (38) | 35 (26) | **0.008** |  |
| Dialysis | 90 (14) | 32 (23) | **0.006** |  |
| PaO_2_/FiO_2_ (n=740) | 172 [101; 284] | 128 [83; 210] | **<0.001** |  |
| VA-ECMO | 9 (1.4) | 2 (1.5) | 1 |  |
| Days in ICU | 3.5 [1.3; 7] | 4 [1.3; 9.6] | 0.085 |  |
| D3 non-survivors | 274 (41) | 60 (44) | 0.63 |  |
| D30 non-survivors | 412 (62) | 102 (74) | **0.006** |  |

Values are expressed as median [25^th^; 75^th^ IQR] or n (%). *Calculated as in [18]. BMI, body mass index; D, day; GCS, Glasgow coma scale; ICU, intensive care unit; SAPS, Simplified Acute Physiology Score; VA-ECMO, venoarterial extracorporeal membrane oxygenation; VIS, vasoactive-inotropic score.
